# Acute Oxygen Consumption Response to Fast Start High-Intensity Intermittent Exercise

**DOI:** 10.3390/sports11120238

**Published:** 2023-12-01

**Authors:** Payton Miller, Noah Perez, John W. Farrell

**Affiliations:** Clinical Biomechanics and Exercise Physiology Laboratory, Texas State University, San Marcos, TX 78666, USA; pem43@txstate.edu (P.M.);

**Keywords:** high-intensity intermittent exercise, critical power, W prime, cycling

## Abstract

The current investigation compared the acute oxygen consumption (VO_2_) response of two high-intensity interval exercises (HIIE), fast start (FSHIIE), and steady power (SPHIIE), which matched w prime (W’) depletion. Eight cyclists completed an incremental max test and a three-minute all-out test (3MT) to determine maximal oxygen consumption (VO_2max_), critical power (CP), and W’. HIIE sessions consisted of 3 X 4 min intervals interspersed by 3 min of active recovery, with W’ depleted by 60% (W’target) within each working interval. SPHIIE depleted the W’target consistently throughout the 3 min intervals, while FSHIIE depleted the W’target by 50% within the first minute, with the remaining 50% depleted evenly across the remainder of the interval. The paired samples *t*-test revealed no differences in the percentage of training time spent above 90% of VO_2max_ (PT ≥ 90% VO_2max_) between SPHIIE and FSHIIE with an average of 25.20% and 26.07%, respectively. Pairwise comparisons indicated a difference between minute 1 peak VO_2_, minute 2, and minute 3, while no differences were present between minutes 2 and 3. The results suggest that when HIIE formats are matched based on W’ expenditure, there are no differences in PT ≥ 90% VO_2max_ or peak VO_2_ during each interval.

## 1. Introduction

For the past century, high-intensity intermittent exercise (HIIE), characterized as repeated bouts of high-intensity exercise separated by periods of recovery, has been observed to be an effective method of exercise training to induce physiological adaptations and increases in human performance [1,2]. The implementation of HIIE in a structured exercise training program has been shown to induce increases in anaerobic and oxidative capacity via oxidative muscle fiber accumulation and capillary density, mitochondrial biogenesis, energy pathway enzyme availability, and increases in cardiac contractile capacity due to myocardium size and stroke volume [3,4]. Researchers have observed that the manipulation of variables, such as number of intervals, work–recovery ratio, and accumulation of work performed, can be used to target anaerobic and oxidative adaptions [1,5].

One of the primary adaptations that endurance athletes target via exercise training is increases in maximal oxygen consumption (VO_2max_) [6,7,8]. Improvements in VO_2max_ are strongly associated with the accumulation of training time spent at exercise intensities equivalent to ≥90% VO_2max_. HIIE has been shown to be an effective training tool in achieving higher intensities; thus, a primary objective when constructing HIIE parameters is to create intervals that allow for the greatest accumulation of time ≥90% VO_2max_ [2]. Previous investigations have observed increases in VO_2max_ following HIIE training primarily related to increases in red blood cell mass, total hemoglobin, blood volume, venous return, end diastolic volume, and ejection fraction [3,9,10].

HIIE has commonly been prescribed using traditional measures such as percent of peak power output (PPO) or maximal velocity associated with VO_2max_, percent of maximal heart rate (HR_max_), percent of gas exchange threshold (GET), or rating of perceived exertion (RPE) [2,11,12]. However, current research supports the significant importance of critical power (CP) as a threshold that demarcates the transition from the heavy- to severe-exercise intensity domains and is considered the new gold standard as a fatigue threshold marker [13]. In conjunction, W prime (W’) establishes the amount of work that can be performed within the severe exercise intensity domain, which is established as above CP [14,15,16,17,18]. When exercising above CP, VO_2_ fails to level off even when the power output is maintained. A metabolic steady state is unachievable; therefore, VO_2_ will continue to drift upwards towards VO_2max_ until the work rate is lowered or volitional exhaustion is reached. Thus, it is crucial to investigate how to best use CP and W’ for prescribing HIIE exercise within the severe-exercise intensity domain.

Previous investigations have examined the use of a fast start, constant power, and decreasing power formats with HIIE and the effects that these have on the priming of VO_2_ kinetics, mean VO_2_ response, and peak power output [14,19]. It was hypothesized that by beginning the initial portion of the working interval at a higher power output before decreasing for the latter portion of the work interval, a VO_2_ priming effect, or rapid increase of VO_2,_ would be induced and sustained despite the subsequent decrease in power output for the latter portion of the working interval. Bailey et al. (2011) found that the fast start pacing strategy led to a faster VO_2_ response, as well as higher mean power during an end sprint compared to a constant power and decreasing power strategy [14]. This agrees with Rønnestad et al. (2022), in which cross-country skiers had a higher percentage of time near VO_2max_, with a fast start compared to constant velocity [19]. However, both of these investigations failed to match intervals based on the depletion of W’. It is possible that by applying the individualized metrics of CP and W’, HIIE protocols following a fast start strategy may result in greater improvements in athletic performance. Based on the findings of the fast-start strategy and VO_2_ response, this study utilized CP and W’ to set HIIE parameters in the fast-start high-intensity interval (FSHIIE) and steady-power high-intensity interval (SPHIIE) conditions to examine the acute physiological responses.

The purpose of this study was to compare the acute physiological responses of varying power output HIIE conditions which were organized as either FSHIIE or SPHIIE and matched on the depletion of W’ by 60% in each working interval. It was hypothesized that the FSHIIE condition would elicit a greater percentage of training time spent above 90% of VO_2max_ (PT ≥ 90% VO_2max_) compared to the SPHIIE condition.

## 2. Materials and Methods

Subjects: Eight trained cyclists completed the investigation. All participants underwent a telephone screening to determine eligibility. Inclusion criteria comprised the following: age between 18 and 64 years, completed planned, structured vigorous-intensity physical activity for ≥30 min at least 3 days per week, reported no cardiovascular, pulmonary, neuromuscular, or neurological conditions or diseases, not currently taking any cardiovascular or respiratory medications, asymptomatic for cardiovascular disease (determined from phone screening tool and Physical Activity Readiness Questionnaire + (PARQ+), able to participate in exercise testing or training without physician’s clearance, considered “low-risk” for the presence of cardiovascular disease or the occurrence of a cardiovascular event occurring during exercise and confirmed normotensive blood pressure, which was assessed on Visit 1 prior to exercise testing [20]. Each participant was informed of the benefits and risks of the investigation prior to signing informed consent, which was obtained from all participants. This study was approved by the Institutional Review Board of Texas State University (IRB #8340). Sample size was selected by convenience and based on previous investigations on PT ≥ 90% VO_2max_ via manipulation of HIIE, with number of participants ranging from 7 to 15 [21,22,23,24,25].

Study Design: The current investigation utilized a cross-sectional, multisession study design to compare the physiological responses to two different HIIE methods performed using an electronically braked cycle ergometer (Lode Excalibur Sport, Lode, Groningen, The Netherlands). Participants were instructed to avoid strenuous exercise 48 h before each visit, not to change their diet while enrolled in this study, and to be at least 2 h postprandial prior to visiting the laboratory. Each visit was separated by at least 48 h and no more than 7 days from the previous testing day. Height and weight were assessed at the beginning of every visit prior to exercise. Participants wore a heart rate monitor (Polar Electro Oy, Finland) for continuous monitoring of heart rate response during all exercise tests. Inspired and expired gases were collected (7450 Series Silicone V2 Oro-Nasal Mask, Hans Rudolph, Shawnee, KS, USA) and analyzed via a calibrated open circuit spirometry system (TrueOne 2400, Parvo Medics, Sandy, UT, USA) during all exercise tests. The gas analyzers were calibrated with room air and gases of known concentration before all testing sessions. Visit 1 consisted of the informed consent, incremental cycling test (ICT), immediately followed by a square wave bout verification protocol. Participants then rested for 30 min before completing the 3-Minute All-Out Test (3MT). Visit 2 consisted of a repeat of the 3MT using the same parameters and instructions as Visit 1. Visits 3 and 4 consisted of a standardized warmup and completion of either FSHIIE or SPHIIE. The order in which HIIE format was completed was randomized, and participants were blinded to the format that was being completed.

### 2.1. Visit 1

Incremental Cycling Test (ICT): Participants gave written consent and completed the physical activity rating scale (PA-R), which was used to determine the starting power for the ICT [26]. The following equations were used to estimate the participant’s VO_2max_ and Peak Work Rate in Watts (W) in order to determine the Work Rate (W) Increments for the ICT [27]:

Estimated VO_2max_ = (56.3) + (1.921 × PA-R) − (0.381 × AGE) − (0.754 × BMI) + (10.987 × Sex); (Sex, 1 = Male, 0 = Female)

Estimated Peak Work Rate = ((Estimated VO_2max_ − 7) × Body Mass)/1.8))/6.12

Work Rate (Watts (W)) Increments = Estimated Peak Work Rate/10 (number of stages)

The cycle ergometer was adjusted to the comfort of each participant, with setting being recorded and replicated for subsequent visits. Participants wore a heart rate monitor (Polar Electro Oy, Kempele, Finland) for continuous monitoring of heart rate response during exercise. Prior to testing, participants were instructed to select and maintain their preferred pedaling cadence throughout all cycling exercises. A >10 RPM decrease in cadence from selected cadence for >5 s, despite verbal encouragement, was indicative of volitional exhaustion and test termination. The ICT began at the predetermined W and increased by the calculated work rate increment every minute until volitional fatigue was reached. To account for the lag in the VO_2_ kinetics during the ramp test, two-thirds of the ramp rate (i.e., 2/3 × 30 W recorded PPO = 20 W) was deducted from the highest power output achieved and was defined as the peak power output (PPO) [28,29,30,31,32]. Upon reaching volitional fatigue, participants continued to exercise at 50 W for a 3 min period of active recovery before completing a verification protocol [31,33,34,35]. At the end of the 3 min period, the exercise intensity was increased to 105% of PPO, and participants cycled at the same cadence as the ICT until volitional exhaustion (i.e., >10 RPM decrease in cadence). Once volitional exhaustion was reached, participants began a 5 min cool-down at 25 W before dismounting cycle ergometer. VO_2max_ was defined as the highest 20 s average obtained during either the ICT or Verification Protocol, and Heart Rate max (HRmax) was defined as the highest 1 s value recorded.

Three-Minute All-Out Test (3MT): Following the 5 min cooldown, participants were given 25 min of rest (total of 30 min of recovery) after completing the ICT and verification protocol before completing the 3MT. Using the data collected from the ICT and verification protocol, parameters for the 3MT were set using the gas exchange threshold (GET) and PPO [36,37]. The GET and PPO were used to normalize the fixed resistance for the 3MT [38]. The resistance for the 3MT was applied using the linear factor function of the cycle ergometer and was calculated with the following equations [39]:

Estimated GET = (PPO/PA-R score^) ^ if PAR > 8 then 0.65, if <8 then 0.60

Estimated 50% Delta = (((PPO − Estimated GET) × 0.5) + Estimated GET)

Torque for 3MT = (Estimated 50% Delta/9.8)

Linear Factor = ((Torque for 3MT/BM (kg))/9.8)

The 3MT protocol began with a warm-up consisting of 3 min of unloaded cycling. During the last 5 s of the warm-up, the participants were instructed to increase their cadence to 110 to 120 rpm. The 3MT involves cycling at all-out effort for 3 min; therefore, instructions were given to reach peak power output as quickly as possible and to maintain the all-out effort throughout the test. Strong verbal encouragement was given throughout the 3MT, and participants were not informed of the elapsed time to prevent self-pacing. Upon completion of the 3MT, participants began a 5 min cool-down at 25 W before dismounting the cycle ergometer. Additional time was provided for the participant to cool down upon request.

### 2.2. Visit 2

The 3MT on Visit 1 served as a familiarization for the 3MT conducted on Visit 2, as the 3MT involves all-out effort without any pacing strategy. This may be unfamiliar to some participants; therefore, the data used to create the parameters for the HIIE programs were from Visit 2 3MT. Height and weight were collected before participants began the 3MT. Visit 2 consisted of a repeat of the 3MT using the same parameters (linear factor) and instructions from Visit 1. VO_2max_ and power data were collected during the test and used to calculate CP and W’ parameters.

#### Calculating Critical Power (CP) and W Prime (W’)

The 3MT has been found to be a valid assessment of CP and W’ quantities [40,41]. Using the 3MT, CP is calculated as the mean power output over the final 30 s of the test, while W’ is calculated as the work performed above CP over the first 150 s of the test. The following equation was used to find W’ from the 3MT:

W’ = ((150 × (p150 − CP))/1000)

Power data from the 3MT included max power achieved (pMax), average power over the first 150 s (p150), and the average power over the last 30 s (CP), which was then used to calculate W’.

### 2.3. Visits 3 and 4

Visits 3 and 4 consisted of either the FSHIIE or SPHIIE session. The order in which HIIE format was to be completed was randomized, and participants were blinded to which format was being assessed. Height and weight were collected before participants were instructed to sit in a chair to obtain their resting blood lactate level. Blood lactate measurements were taken from the fingertip using a lancet (Unistik 3, Owen Mumford Ltd., Brook Hill, Wood Stock, Oxfordshire, UK) and a lactate strip (Lactate Plus, Nova Biomedical, Waltham, MA, USA) and analyzed using a lactate analyzer (Lactate Plus, Nova Biomedical, Waltham, MA, USA). Once resting blood lactate was recorded, the participants began the assigned HIIE protocol.

Each HIIE session began with a 15 min standardized warmup. Participants began with 5 min of cycling at 50 W, followed by 5 min of cycling at each participant’s GET, and then 5 min at 50 W. Immediately after completing the warmup, the first interval began. Both the FSHIIE and SPHIIE intervals were designed to deplete W’ by 60% (W’target) in each working interval [41,42,43,44,45]. The time intervals followed previous recommendations of performing 4 and 3 min intervals (4 X 3 min) with 3 min of active recovery at 50 W between each high-intensity interval (1:1 work-to-rest ratio) [13,41,46,47,48]. The only difference between sessions was the amount of the W’target that was depleted in each minute of the intervals. FSHIIE was designed to deplete 50% of the determined W’target within the first minute of the 3 min interval, with the remaining 50% evenly dispersed between minutes 2 and 3 (25%, respectively). For example, if a participant had a W’ of 10 kJ, this would result in a W’target of 6 kJ. Within the first minute of the FSHIIE session, 3 kJ of work would be completed (50% of W’target) with 1.5 kJ of work performed in minutes 2 and 3 (25% of W’target each). This interval setup was repeated for the entire 4 intervals completed. SPHIIE was designed to deplete the determined W’target evenly across the 3 min interval (33.33%, respectively). Therefore, in both HIIE sessions, the same amount of W’target was depleted, with varying power in FSHIIE and constant power in SPHIIE. The following equation was used to determine the work rate for each interval:

(W’ to be depleted/desired length of interval) + Critical Power = Interval work rate (Watts)

Both formats followed a 1:1 work-to-rest ratio, allowing 3 min of active recovery at 50 W between each work interval. This has been shown to allow for the repletion of W’ between intervals [49,50,51,52]. During each HIIE session, inspired and expired gases were collected continuously with rating of perceived exertion (RPE), using the Borg Scale, and blood lactate was assessed immediately after each work interval (i.e., after the completion of each 3 min high-intensity interval) [53]. Upon completion of the final high-intensity interval, participants completed 3 min of active recovery at 50 W and then immediately completed a 5 min cool-down at 25 W.

### 2.4. Statistical Analysis

All analyses were performed using IBM SPSS Statistics (version 26.0; IBM Corp., Armonk, NY, USA). Descriptive statistics were used to summarize the demographic data. VO_2_ data were exported in 5 s averages and used for analysis. Data from each minute of the intervals were averaged within each trial (e.g., the minute 1 VO_2_ response from each of the four work intervals in the FSHIIE were averaged, and the same for minutes 2 and 3 and the SPHIIE [19]. Peak VO_2_ was defined as the highest VO_2_ response in each minute of the work interval and was also expressed as a percentage of VO_2max_. A two-way repeated measure analysis of variance (time × condition) was used to detect differences in VO_2_ response between minutes 1, 2, and 3 of each condition. Bonferroni post hoc analyses were conducted if significant interactions were detected. Eta squared (η^2^) was used to interpret effect sizes for the ANOVA, with values of 0.02, 0.13, and 0.26 indicative of small, medium, and large effects, respectively. RPE and blood lactate were averaged across the 4 intervals within each session with peak RPE and peak blood lactate defined as the highest value obtained in each session. Additionally, the PT ≥ 90% VO_2max_ was calculated for each session. Paired sample *t*-tests were used to detect statistically significant differences between FSHIIE and SPHIIE in terms of RPE, blood lactate, and PT ≥ 90% VO_2max_. Cohen’s *d* effect sizes were utilized, with values of 0.2, 0.5, and 0.8 indicating small, medium, and large effects, respectively.

## 3. Results

A total of eight participants (*n* = 8; 7 males, 1 female) completed the investigation, and individual data are reported in Table 1. According to De Pauw et al.’s 2013 guidelines for the performance classification of cycling-related research, the current cohort consists of two, five, and one performance levels and relative VO_2max_ values of two, three, and four, respectively [54].

**Figure 1:**
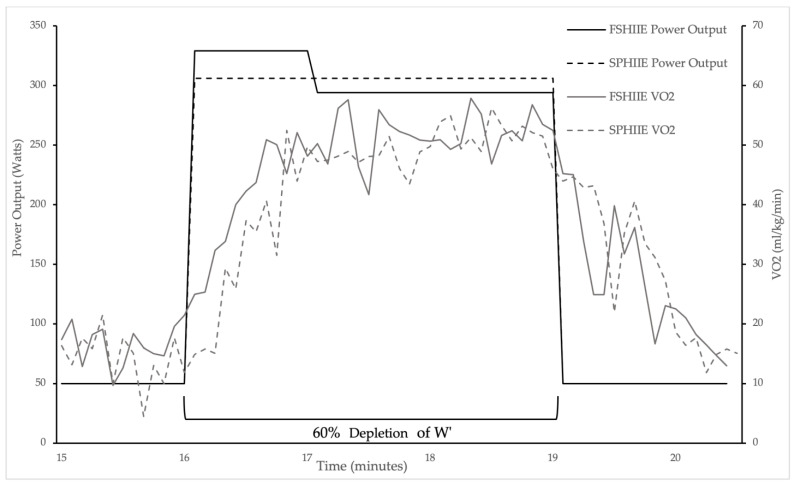
Representative individual oxygen consumption response to both fast-start and steady-power high-intensity intermittent exercise.

Data pertaining to oxygen consumption responses to HIIE are reported in Table 2. The repeated measure ANOVA revealed no statistically significant condition × time interaction (f = 0.482, *p* = 0.628, and η = 0.064) for the average VO_2_ response with no statistically significant main effect for the condition (f = 1.669, *p* = 0.237, η = 0.192). However, a significant main effect for time was present (f = 50.932, *p* = 0.000, η = 0.879). Pairwise comparisons indicated a statistically significant difference between minute 1 and minute 2 (*p* = 0.001, *d* = 2.9), minute 1 and minute 3 (*p* = 0.000, *d* = 3.31), and minute 2 and minute 3 (*p* = 0.014, *d* = 0.29). The repeated measure ANOVA revealed no statistically significant condition × time interaction (f = 0.093, *p* = 0.912, and η = 0.013) for the average peak VO_2_ response, with no statistically significant main effect for the group (f = 0.244, *p* = 0.636, η = 0.034). However, the significant main effect for time was present (f = 77.692, *p* = 0.000, η = 0.917). Pairwise comparisons indicated a statistically significant difference between minute 1 and minute 2 (*p* = 0.000, *d* = 1.69) and minute 1 and minute 3 (*p* = 0.000, *d* = 2.05), with no significant difference between minute 2 and minute 3 (*p* = 0.069, *d* = 0.23). Figure 1 illustrates a representative individual’s VO_2_ response to both FSHIIE and SPHIIE.

Acute perceptual and blood lactate responses to HIIE are reported in Table 3. Paired sample *t*-tests revealed no significant differences for peak (t = 1.080, *p* = 0.316, *d* = 0.3) and average (t = 0.523, *p* = 0.617, *d* = 0.1) RPE and peak (t = −1.220, *p* = 0.262, *d* = 0.4) and average (t = −1.057, *p* = 0.326, *d* = 0.3) lactate between conditions. The paired sample *t*-tests revealed no significant differences for PT ≥ 90% VO_2max_ between the conditions (t = −0.159, *p* = 0.878, *d* = 0.01).

## 4. Discussion

The main purpose of this study was to investigate the physiological responses to two different HIIE conditions, which were matched based on the depletion of W’ by 60% in each working interval. The primary hypothesis was that the FSHIIE condition would elicit a greater PT ≥ 90% VO_2max_ compared to the SPHIIE condition. The null hypothesis was accepted, as there were no significant differences found between the two conditions.

Previous investigations have used both CP and W’ to prescribe HIIE and compare different HIIE formats. However, the current investigation is the first to explore the use of fast start intervals when matched based on W’ expenditure. Appropriate external work rates or power outputs for working intervals of HIIE can easily be calculated using the desired W’ depletion across a given duration. Theoretically, the same metabolic strain can be applied across varying durations as long as the depletion of W’ is matched. A recent investigation observed a lower adherence, 20%, to HIIE when prescribed using VO_2max_ when compared to HIIE prescribed using CP and W’, 100% [55]. It was hypothesized that this was due to the traditional measures groups’ inability to demarcate the heavy- to severe-intensity domains within their participants, which leads to higher variability compared to when using CP [55]. Using CP and W’ metrics allowed this study to match work intervals in the severe exercise-intensity domain [56,57,58]. By implementing CP and W’, we hypothesized that the HIIE sessions can be better tailored to increase athletic performance [43].

The current investigation matched the working intervals via W’ depletion while applying varied power outputs between conditions. Again, this differs from the most common application of HIIE interval parameters, which have followed a method of constant work rate in the interval followed by a rest period. A plethora of studies have investigated the outcomes of low-, moderate-, and high-intensity intervals, yet the use of varied power outputs within the working intervals has been limited. However, a study by Rønnestad et al. (2022) implemented the use of a fast-start strategy in which a group of cross-country skiing participants began their high-intensity interval at a higher power output (based on maximal aerobic speed and velocity that elicited lactate threshold response) for 2 min, which then dropped in power for the remaining 3 min of the interval [19]. This study compared three different conditions and found that the fast-start group and varied power output group led to a higher PT ≥ 90% VO_2max_ compared to a constant power interval group. A limitation of the investigation by Rønnestad et al. (2022) was the use of the TM to set the HIIE parameters that do not account for the demarcations between exercise intensity domains [19].

Within the current investigation, no significant differences were detected in PT ≥ 90% VO_2max_. This is due to matching the depletion of W’ by 60% in each working interval for both conditions. By matching W’, participants were completing the same amount of work in kjs and time above their CP in both HIIE conditions, which would, therefore, lead to similar VO_2_ responses. Although there were no differences found between groups in PT ≥ 90% VO_2max_, participants completed the SPHIIE and FSHIIE sessions with an average of 25.20% and 26.07% of PT ≥ 90% VO_2max_, respectively. According to a systematic review by Buchheit and Laursen (2013), the PT ≥ 90% VO_2max_ found during running intervals based on the velocity of VO_2max_ ranging from 2 min to 5 min intervals were 13%, 19%, 21%, and 26% [2,10]. Based on these data, the FSHIIE and SPHIIE conditions were quantified for greater training time near VO_2max_. It can then be hypothesized that when utilizing W’ or D’ (distance prime) to set HIIE parameters, a greater PT ≥ 90% VO_2max_ can be achieved. Beneficially, when training time is near VO_2max_, there will also be an increase in VO_2max_, CP, and the aforementioned physiological adaptations. This subsequently will lead to improvements in both athletic performance and health status.

There was a statistically significant difference between the VO_2_ response from minute 1 compared to minute 2 and minute 3 in the working intervals. The observed rapid increase in VO_2_ kinetics at the initiation of the work interval is known as the fast component, also known as phase 1 of the gas exchange phases of VO_2_ kinetics. This phase occurs when there is an initiation of exercise with a need to increase oxygen uptake to provide the working muscles with oxygen for oxidative phosphorylation to continue power output [59,60]. During phase 1, the rapid increase in VO_2_ is also due to the large amount of CO_2_ production that is associated when utilizing anaerobic energy pathways [61]. When participants transitioned from 50 W to their individual interval power outputs, the VO_2_ time constraint, which is known to exponentially increase VO_2_ to match the ATP requirement, caused the large slope seen within minute 1 VO_2_. The participants in this study were trained cyclists, which also accounts for the accelerated phase 1 as well as increases in the priming of mitochondrial enzymes [62,63]. With an improved ability to distribute and extract O_2_ in the blood to localized muscle, a large increase would be seen in the first minute with a leveling off in the subsequent minutes of the working intervals, if they were at a constant work rate that was below CP [59,64]. In this case, however, participants were set above CP; therefore, there would be no leveling off, but a slight increase in VO_2_ until power output was lowered.

There were no differences found between the minute 2 and minute 3 VO_2_ responses, which is due to the slow component, also known as phase 2, of VO_2_ kinetics. After phase 1 occurred within minute 1, which allowed for the blood flow distribution and extraction of O_2_ in the working muscles, a slowly developing VO_2_ took place over minute 2 and minute 3 [63,65]. While not statistically different, there was no leveling off of VO_2_ found within these minutes as the steady state cannot be reached when exercise intensity is above CP within the severe exercise intensity domain [11,59]. A hallmark characteristic of exercise within the severe-exercise intensity domain is the continuous drifting of VO_2_ upwards towards VO_2max_. The relationship between W’ depletion and the development of the slow component can be attributed to the increased recruitment of type II muscle fibers due to muscle fatigue in type I that leads to less efficient use of O_2_, and the reliance on phosphocreatine as well as fatigue due to H^+^ accumulation [59,65,66,67]. Endurance athletes have been found to have a relatively smaller slow component phase as this population has CP levels that are near their VO_2max_ due to the physiological adaptations mentioned previously [59]. Therefore, no differences were found between minute 2 and minute 3 VO_2_ as the VO_2_ response would slowly drift upwards until power output dropped below CP, or volitional exhaustion was reached.

It is interesting that the FSHIIE group had no significant differences from the SPHIIE group considering that the FSHIIE condition dropped to a power output that was lower for the last 2 min of the intervals. Reasonably, it would be hypothesized that the drop in power below the SPHIIE condition would reflect differences in the VO_2_ response. However, this was not the case, as no differences were found. It can be speculated that the combined results are due to not accounting for the severe exercise intensity zones that the participants were completing the intervals in [68]. Within the severe exercise intensity domain, there is zone 1 (Z1), which is found above CP, zone 2 (Z2), which is above Z1 at approximately 50% of the difference between CP and VO_2max_ power, and the extreme zone (EZ) [68,69]. When the exercise is conducted above CP, the slow component of VO_2_ response will be dependent on the severe intensity zone, while peak VO_2_ cannot be obtained within the EZ [69,70]. This study did not establish the Z1, Z2, or EZ within the participant’s intervals; therefore, a discrepancy in individual exercise intensity could have taken place. Without constituting the participants in matched intensity zones, participants with smaller W’s could have been in a higher zone compared to those with a larger W’. In relation to the fast and slow components of the VO_2_ response and PT ≥ 90% VO_2max_, if matched within Z1 or Z2 for all participants, differences might have been found.

Other limitations in this study include matching the work-to-rest ratios, as well as matching the percentage of W’ depletion between the FSHIIE and SPHIIE conditions. Previous investigations have manipulated the time of the working intervals from short sprints (5–10 s) up to 3 min as well as decreasing the rest intervals allowed for adaptations of the neuromuscular, anaerobic, and aerobic systems and increases in time spent near VO_2max_ [2,7,10,71]. This, however, has not been tested when using CP and W’ to set HIIE parameters. Future research is needed to implement CP and W’ in HIIE conditions that modify the interval times, work-to-rest ratio, and/or the adjustment of the percentage of W’ depletion in each interval as well as testing different populations.

## 5. Conclusions

In conclusion, trained cyclists had no significant difference in PT ≥ 90% VO_2max_ between the FSHIIE and SPHIIE conditions when W’ depletion is matched. Applying a fast-start, varied power output method using CP and W’ to HIIE intervals could increase the priming and fast component of VO_2_ kinetics as well as better prepare athletes for race scenarios. This accounts for road cyclists’ race performance needs that include increases and decreases in power output depending on the terrain, team strategy, and individual performance. Therefore, by applying a fast-start strategy compared to a constant power, the racing conditions could be better applied. Further studies are needed in order to investigate the acute physiological differences, chronic cardiovascular and metabolic adaptations that can be observed when HIIE sessions are curated with CP and W’, and the manipulation of the above variables.

## Figures and Tables

**Table 1:** Individual demographic data.

Participant	Sex	Age (years)	Height (cm)	Body Mass (kg)	VO_2max_ (mL∙kg^−1^∙min^−1^)	CP (W)	W’ (Kj)
1	M	19	173	65.70	66.30	304.50	11.26
2	M	31	176	76.00	55.50	291.85	10.92
3	M	20	166	60.00	64.60	289.81	13.55
4	M	27	173	94.90	45.30	289.27	10.15
5	M	29	174	68.10	55.00	259.29	14.05
6	M	28	179	77.50	59.60	339.72	19.06
7	F	36	158	58.20	55.20	242.89	10.98
8	M	24	183	102.80	53.50	341.39	12.68

Abbreviation: M = male, F = female VO_2max_ = maximal oxygen consumption, CP = critical power, W’ = W prime, W = watts, Kj = kilojoules.

**Table 2:** Oxygen consumption response across intervals.

	Conditions											
	SPHIIE			FSHIIE			Main Effect Condition		Main Effect Time		Condition X Time Interaction	
	Time (Minutes)			Time (Minutes)								
Variable	1	2	3	1	2	3	*p*	η^2^	*p*	η^2^	*p*	η^2^
Average VO_2_ (mL∙kg^−1^∙min^−1^)	31.39 (4.28)	51.59 (5.77)	53.96 (5.36)	29.96 (5.37)	47.84 (11.94)	50.07 (11.66)	0.237	0.192	0.000 *	0.879	0.628	0.064
Average Peak VO_2_ (mL∙kg^−1^∙min^−1^)	42.45 (5.59)	53.64 (6.00)	55.01 (5.47)	41.61 (7.63)	52.88 (8.38)	54.68 (7.33)	0.636	0.034	0.000 *	0.917	0.912	0.013

Data are presented as mean (SD). Abbreviations: VO_2_ = oxygen consumption; SPHIIE = steady power high-intensity intermittent exercise; FSHIIE = fast start high-intensity intermittent exercise; η^2^ = effect size, expressed as partial eta squared. * *p* < 0.05 represents statistical significance.

**Table 3:** Acute perceptual and blood lactate responses to high-intensity intermittent exercise conditions.

Variable	FSHIIE	SPHIIE
% of Total Training Time Above 90% VO_2max_	25.20 ± 8.77	26.07 ± 11.84
Peak Lactate	14.77 ± 3.43	16.02 ± 2.39
Average Lactate	12.98 ± 2.28	13.61 ± 1.55
Peak RPE	19.00 ± 1.30	18.50 ± 1.69
Average RPE	17.21 ± 1.26	17.06 ± 1.74

All data are presented as mean ± standard deviation (SD) %: percent, VO_2max_: maximal oxygen consumption (mL∙kg^−1^∙min^−1^), RPE: rate of perceived exertion.

## Data Availability

The data presented in this study are available upon request from the corresponding author.
